# Cost-Effectiveness Analysis of Group vs. Weblog Telecommunication (Web Tel) Nutrition Education Program on Glycemic Indices in Patients With Non-Insulin Dependent Diabetes Mellitus Type 2: A Randomized Controlled Trial

**DOI:** 10.3389/fnut.2022.915847

**Published:** 2022-06-24

**Authors:** Seyedeh-Masomeh Derakhshandeh-Rishehri, Khosro Keshavarz, Delaram Ghodsi, Gholamreza Pishdad, Shiva Faghih

**Affiliations:** ^1^Department of Community Nutrition, School of Nutrition and Food Sciences, Shiraz University of Medical Sciences, Shiraz, Iran; ^2^Health Human Resources Research Center, Department of Health Economics, School of Management and Information Sciences, Shiraz University of Medical Sciences, Shiraz, Iran; ^3^Department of Nutrition Research, National Nutrition and Food Technology Research Institute, Faculty of Nutrition Sciences and Food Technology, Shahid Beheshti University of Medical Sciences, Tehran, Iran; ^4^Department of Internal Medicine, Endocrinology and Metabolism Research Center, Shiraz University of Medical Sciences, Shiraz, Iran

**Keywords:** diabetes, nutrition, education, cost-effectiveness, Web

## Abstract

This a randomized controlled trial study with a cost-effectiveness analysis that aimed to compare the cost-effectiveness of group nutrition education with that of Web-Tel nutrition education in the glycemic control of patients with non-insulin-dependent type 2 diabetes mellitus (T2DM). The study was conducted on 105 patients with T2DM for 3 months in Quds health centre of Bushehr province, Iran. The participants were classified based on age and disease severity (hemoglobin A1c level); then, they were randomly assigned to one of the three groups: group education, Web-Tel education, and the control group using block randomization method. The clinical (intermediate) outcome was changes in hemoglobin A1c (HbA1c). Patients' perspective was adopted, and a deterministic one-way sensitivity analysis was conducted to identify the effects of uncertainties. The results indicated that the expected effectiveness was 0.46, 0.63, and 0.4; the mean costs was 27,188, 5,335, and 634 purchasing power parity (PPP) dollars for group education, Web-Tel education, and the control group, respectively. The incremental cost-effectiveness ratio (ICER) of Web-Tel education vs. the control group was positive and equal to $21, 613.04 PPP; since it was less than three times of the threshold, the Web-Tel education method was considered as a more cost-effective method than the control group. On the other hand, the ICER of group education vs. control group was $447,067 PPP and above the threshold, so group education was considered as a dominated method compared with the control group. In conclusion, considering the ICER, Web-Tel education is a more cost-effective method than the other two and can be used as the first priority in educating patients with T2DM. The present study was registered in Thailand Clinical Trials Registry (TCTR20210331001).

## Introduction

Uncontrolled diabetes mellitus (DM) could lead to micro-/macrovascular complications, decreasing the quality of life, increasing the risk of premature death, and imposing a substantial economic burden of work absenteeism and healthcare on societies (1–5). The total annual cost of diabetes in Iran is estimated to 3.64 billion US dollars (including $1.71 billion direct and $1.93 billion indirect costs) in 2009, and is estimated to reach to 9 billion US dollars by 2030 (including $4.2 billion in direct and $4.8 billion in indirect costs) (1).

Education is considered as a basic principle in diabetes care (6). Accordingly, one of the most important methods in diabetes self-management education (DSME) is “group education.” This method enables patients to discuss nutritional issues and increase their quality of life (6–8). On the other hand, Web-Tel nutrition education includes the use of weblog and mobile apps, and is considered as a new way for educational purposes (9).

There are numerous studies worldwide that focused on the economic burden of DM, but limited studies have been conducted on the cost-effectiveness of nutrition education in patients with diabetes. A 6-month randomized controlled trial examined the cost-effectiveness of nutrition education in 179 patients with diabetes but free of complications of diabetes and co-morbidities in the United States. The results showed that the cost-effectiveness was $4.2 in the intervention group and $5.32 in the control group (10). Also, three studies were conducted in 2014, 2015, and 2018; all showed that DSME had better cost-effectiveness than usual care in the quality of life (11–13).

Although a group or virtual nutrition education program has been tried on a worldwide scale, the results have been inconclusive. To the best of our knowledge, there is no consensus on the most cost-effective approach of nutrition education for patients with diabetes. Therefore, the present study aimed to compare the cost-effectiveness of two nutrition education methods, Web-Tel and group education, with a control group in the HbA1c level of patients with non-insulin-dependent T2DM.

## Materials and Methods

### Study Design and Participants' Recruitment

The present study was a cost-effectiveness analysis and a parallel randomized controlled clinical trial. The study population consisted of adult patients with T2DM who were recruited from Quds health centre of Bushehr province, Iran in 2021. The inclusion criteria included: patients with T2DM with poor glycemic control (HbA1c ≥ 7), diagnosis of the disease under 1 year, patients with non-insulin-dependent type 2 diabetes (either on medication or not), adults above 20 years old, reading and writing literacy; having access and ability to use the internet and mobile phones, and tendency to participate in the study. However, the following individuals were not included in the study: individuals with any mental or physical disabilities, individuals under treatment for AIDS, cancers, chronic heart failure, cerebrovascular, renal, and hepatic diseases, pregnant or lactating women, individuals who have participated in professional nutrition education classes during the last year, and individuals who attended in other clinical studies in the past 6 months. The exclusion criteria included being diagnosed with any chronic diseases during the trial, failure to attend more than one educational session, and lack of motivation to continue at any time of the study. Before the study commenced, the main researcher explained all the possible benefits and harms of it to the participants, and then written consent forms were signed by them.

### Sample Size Calculation

According to the previous study by Kim et al. (14), and considering HbA1c as a primary outcome, with the power and confidence level of 99%, sample size was calculated as 26 participants in each group using the NCSS (PASS) 2007 software (NCSS, Kaysville, UT, United States). After applying attrition rate of 35% in sample size calculation, a total of 105 participants were obtained (*n* = 35 in each group). The following formula was used for sample size calculation:


$$\begin{array}{l} N=\frac{\;\left(Z_{1-\alpha /2}\;+\;Z_{1-\beta }\right)\left(\delta_{1}^{2}+\;\delta_{2}^{2}\right)}{d^{2}} \end{array}$$

In the above formula: d = 100 z, α = 0.01, β = 0.01, δ_1_ = 0.81, and δ_1_ = 0.61.

### Random Allocation and Allocation Concealment

After checking the participants' compatibility with the inclusion and exclusion criteria, eligible ones received a unique code. In order to control for prognostic factors, the participants were classified based on their age and disease severity (hemoglobin A1c); then, they were randomly assigned to one of the three groups using block randomization method by the main researcher. For this purpose, random allocation software version 2.0 was used.

### Data Collection and Measurement Intervals

At the beginning of the study, a demographic questionnaire (including sex, age, education, and occupation) was completed by each participant. Glycemic indices were measured in months 0 and 3 of the study. The intermediate (clinical) outcome was changes in HbA1c.

### Biochemical Measurements

Participants' blood samples were collected early in the morning after at least 10 h of fasting. Also, they were asked to stop taking glucose-lowering drugs for 8–10 h before the test. HbA1c was measured using a complete blood sample by high-performance liquid chromatography (HPLC) (Bio-Rad Variant II, Sydney, Australia). Fasting blood sugar (FBS) was measured by hexokinase (Roche Modular Analyzer; Tokyo, Japan). Fasting insulin levels was measured quantitatively by enzyme-linked immunosorbent assay (ELISA).

### Intervention

The current study was divided into two stages (2 phases).

#### Phase 1 (Pre-intervention) for 2 Weeks

In this phase, “demographic” questionnaires were completed. In addition, participants in the Web-Tel group were taught how to work on the internet in the form of weblog and WhatsApp by the main researcher at the Quds health centre of Bushehr province.

#### Phase 2 (Intervention) for 3 Months

**Figure 1:**
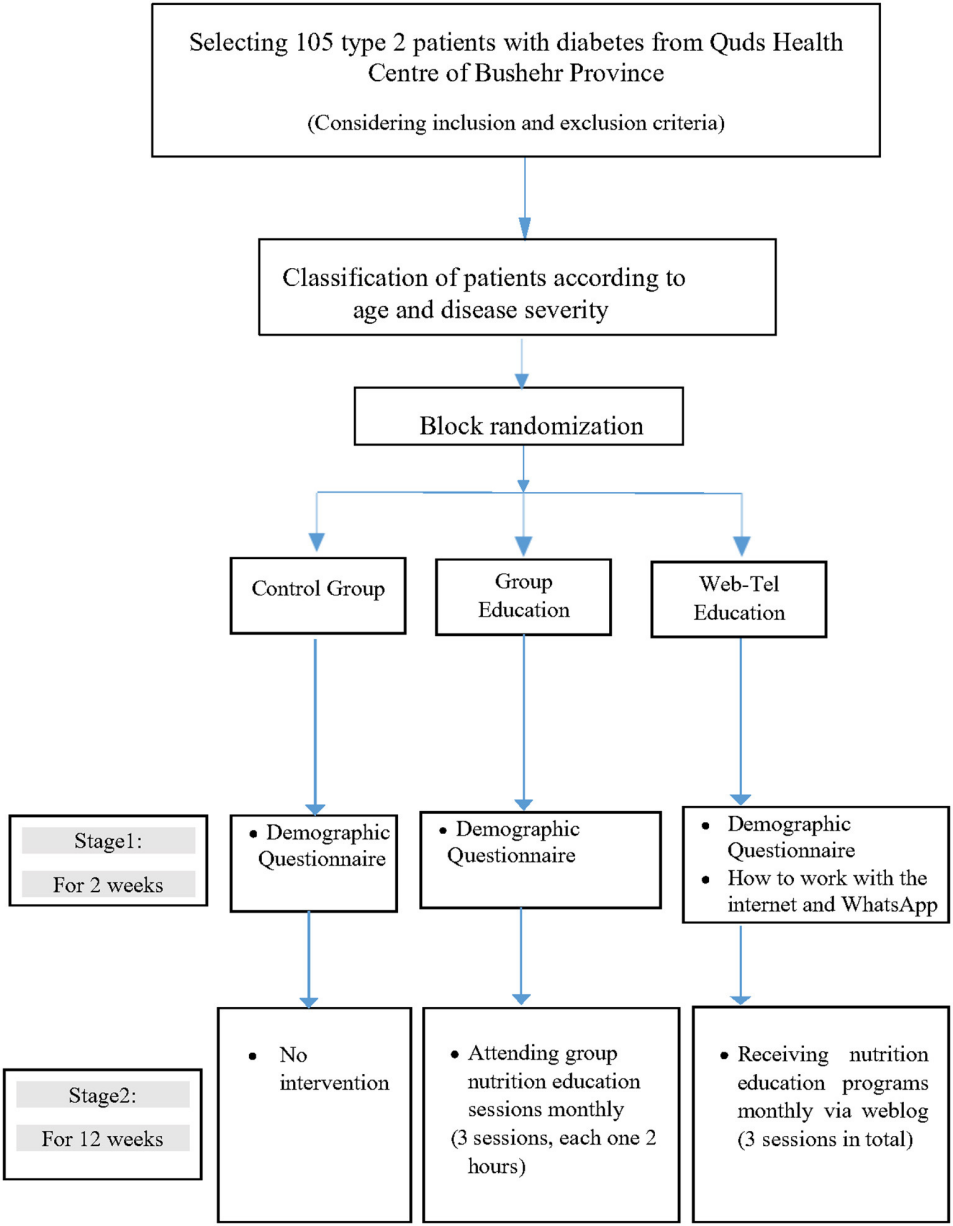
Summary of intervention on patients with type 2 diabetes mellitus (T2DM).

In this phase, the participants received nutrition education. The educational contents were the same in both groups (Web-Tel and group method). The Web-Tel group received one lesson each month. They used their special password to enter the weblog and read the educational contents. In group education, educational sessions were held monthly (3 sessions in total and each session lasted for 2 h). The control group received none of the nutrition educational contents that were delivered to the intervention groups. Due to the COVID 19 pandemic, none of the participants received any usual care. The study design and educational contents are summarized in Figure 1 and Supplementary Table 1, respectively.

### Statistical Analysis

The collected data were analyzed with SPSS software version 22, within an intent-to-treat (ITT) framework in which missing data were simply imputed and the last measurement of every participant was used. Baseline values were shown as mean ± standard deviation (SD) for quantitative variables, and frequency (percentage) for qualitative variables. The chi-square method was used to compare qualitative variables among the three groups. For quantitative variables, first, the normality of the data was assessed by Kolmogorov-Smirnov and Shapiro-Wilk tests. Then, for between-group comparisons of normally distributed variables, one-way ANOVA, and for abnormal distributed ones, Kruskal Wallis test were conducted. *P* < 0.05 was considered as significant.

### Data Collection of Costs

The cost-effectiveness was determined with patient perspective. In general, the costs were divided into two categories: (1) direct medical costs (DMCs) including cost of education, educational tools, and execution, and (2) direct nonmedical costs (DNMCs), which were the costs of infrastructural materials. DMCs and DNMCs were collected using cost collection forms, self-reporting of the patients, and experts' opinions. Details of costs have been summarized in the footnotes of **Table 2**.

### Cost-Effectiveness Analysis

In the present study, effectiveness was defined as a reduction in HbA1c level. Then, costs, effectiveness, and incremental cost-effectiveness ratio (ICER) were calculated with TreeAge Software version 2020. ICER was calculated using the following formula: (cost _intervention_ – cost _control_)/(Effectiveness _intervention_- Effectiveness _control_).

### Uncertainty Analysis

A one-way sensitivity analysis was conducted to assess the effect of the parameter's uncertainty on the results of the study. The parameters included the effectiveness of group education, Web-Tel education, or the control group, and the costs of group education, Web-Tel education, and the control group. The values of each parameter changed by 20%, and tornado diagrams were drawn. Since there is no specific cost-effectiveness threshold in Iran, based on WHO recommendation, the threshold in developing countries was considered at one time to three times of gross domestic product per capita. According to the World Bank report, the threshold was about $13116 PPP for Iran in 2021 (15, 16).^1^,^2^

## Results

In the present study, twenty-three patients discontinued their participation (overall attrition rate = 21.9%). The attrition rate in group education was 22.85%, in Web-Tel education 20%, and in the control group 22.85% (Supplementary Figure 1). Also, the between-group comparisons of baseline variables indicated no significant differences (Table 1). According to Table 2, the mean DMCs per participant in group education, Web-Tel education, and the control group were 194.79, 335.98, and 30.91 dollars, respectively. Moreover, the mean DNMCs per participant in group education, Web-Tel education, and the control group were 26,993.7, 4,998.82, and 333.25 dollars, respectively. Based on the Kruskal-Wallis test, there was a significant difference among the three groups in each category of DMCs, DNMCs, and total costs (*p* < 0.001).

**Table 1:** Baseline characteristics of the participants.

**Variables**		**Group education**				**Web-tel education**				**Control group**				***P*-value**
		**Mean**	**SD**	***N***	**%**	**Mean**	**SD**	***N***	**%**	**Mean**	**SD**	***N***	**%**	
Sex	Male			14	40			13	37.1			11	31.4	0.75^a^
	Female			21	60			22	62.9			24	68.6	
Age		55.40				56.02				50.71				0.22^b^
Medication*				27	77.1			23	65.7			22	62.9	0.39^a^
Education	Primary			9	25.7			9	25.7			7	20	0.77^a^
	Secondary			7	20			5	14.3			7	20	
	High-school/Diploma			11	31.4			10	28.6			7	20	
	University			8	22.9			11	31.4			14	40	
Occupation	Housewife/unemployed			16	45.7			17	48.6			20	57.1	0.05^a^
	Shop worker, simple worker, semi-skilled worker, driver, parttime employee, low ranking military officer			1	2.9			0	0			6	17.1	
	Skilled worker, employer, experienced employee, military with the rank of officer to major			6	17.1			6	17.1			1	2.9	
	Government officials, physicians, university professors, army officers (at least major)			2	5.7			4	11.4			3	8.6	
	Retired			10	28.6			8	22.9			5	14.3	
FBS (mg/dl)		166.31	42.57			160.34	44.05			166.37	51.44			0.82^c^
HbA1c (U%)		9.01	1.72			9.19	2.01			8.49	1.68			0.24^c^
Insulin (μIU/mL)		13.49	7.17			9.90	6.34			11.65	6.28			0.08^c^

*^a^Results are expressed according to chi-square test*.

*^b^Results are expressed according to Kruskal-Wallis test*.

^c^Results are expressed according to one-way ANOVA. ^*^Medications used by the participants in the present study included metformin (glucophage), glibenclamide (glyburide), acarbose, and glipizide.

**Table 2:** Comparison of costs among the three groups of patients with type 2 diabetes mellitus (T2DM).

**Costs**	**Details**	**Group education**		**Web-tel education**		**Control group**		***P*-value**
		**mean**	**%**	**mean**	**%**	**mean**	**%**	
Direct medical costs	Cost of education^a^	$27.14	0.10	$29.11	0.55	0.0	0.0	<0.001^ef^
	Cost of educational tools^b^	$103.72	0.38	$123.51	2.31	0.0	0.0	<0.001^eg^
	Executive costs^c^	$63.93	0.24	$183.36	3.44	$30.91	8.49	<0.001^eh^
Direct non-medical costs	Infrastructurecosts^d^	$26993.70	99.28	$4998.82	93.70	$333.25	91.51	<0.001^eh^
Total costs		$27188.49	100	$5334.80	100	$364.16	100	<0.001^eh^

*^a^Cost of nutrition education for three sessions*.

*^b^Costs of educational tools for three sessions include preparing and uploading of educational contents*.

*^c^Executive costs include costs of calling, messaging, using internet, and creating a weblog*.

*^d^Infrastructure costs include costs of providing a telephone, a place for training, and a computer and setting up a technology platform*.

*^e^Results are expressed according to Kruskal-Wallis test*.

*^f^The results of Mann-Whitney U-test showed significant differences for all comparisons of group education vs. control group (P < 0.001) and Web-Tel education vs. control group (P < 0.001)*.

*^g^The results of Mann-Whitney U-test showed significant differences for all comparisons of group education vs. Web-Tel education (P = 0.003), group education vs. control group (P < 0.001), and Web-Tel education vs. control group (P < 0.001)*.

*^h^The results of Mann-Whitney U-test showed significant differences for all comparisons of group education vs. Web-Tel education (P < 0.001), group education vs. control group (P < 0.001), and Web-Tel education vs. control group (P < 0.001)*.

**Figure 2:**
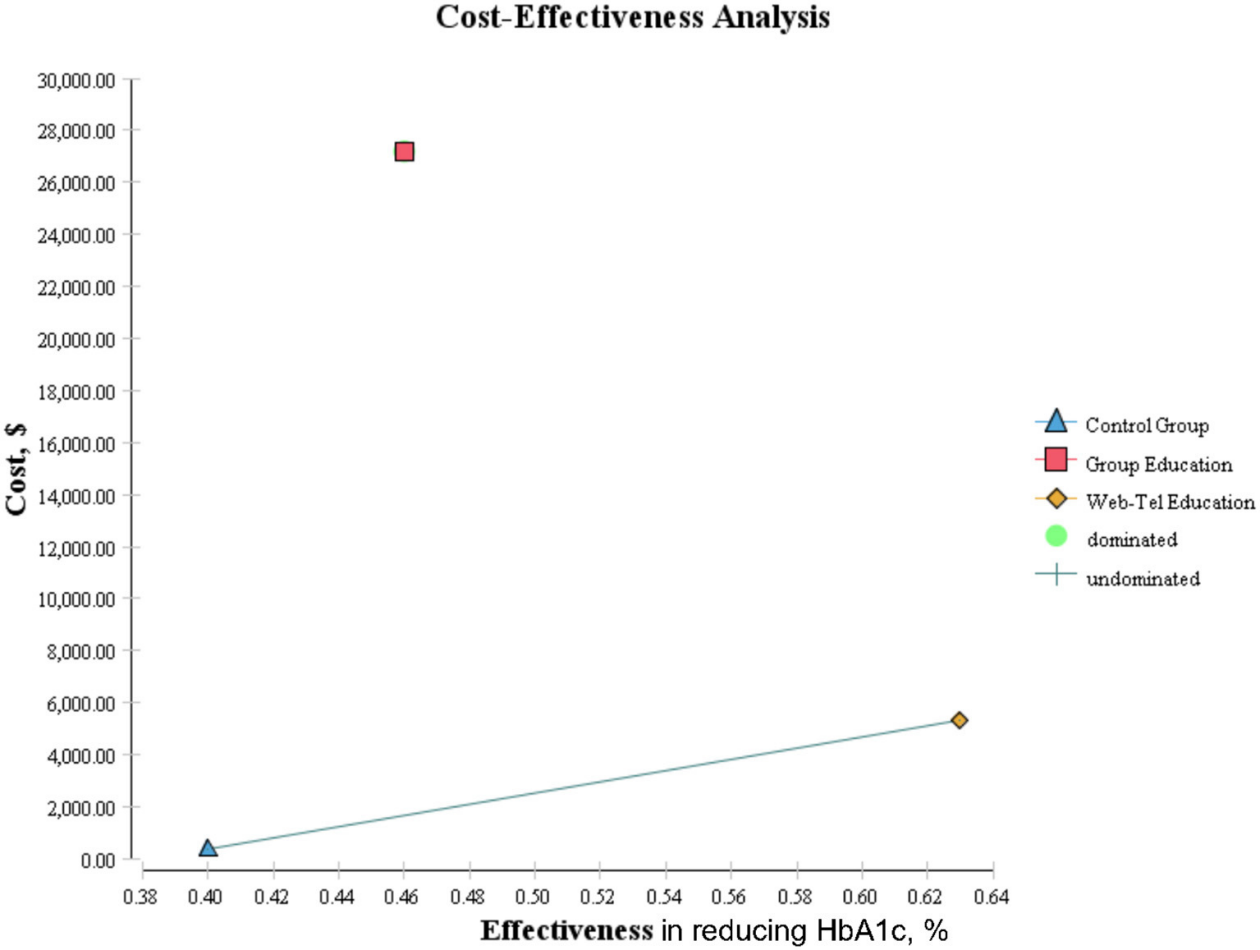
Comparison of cost-effectiveness analysis among three groups of patients with type 2 diabetes.

As shown in Table 3 and Figure 2, the expected effectiveness was 0.46, 0.63, and 0.4; the mean costs were 27,188, 5,335, and 634 PPP dollars for group education, Web-Tel education, and the control group, respectively. The comparison of Web-Tel education vs. control group showed that the ICER was $21,613.04, which indicated that for each additional percentage of success in decreasing HbA1c level by Web-Tel education, $21,613.04 should be spent. On the other side, the comparison of group education vs. control group showed that the ICER was $447,067, which indicated that for each additional percentage of success in decreasing HbA1c level by group education, $447,067 should be spent. To make a decision, we must compare the ICER with a threshold (15). Therefore, because the ICER was less than three times per capita GDP, the Web-Tel method was considered as a more cost-effective option than the control group, while the group education method was more than one to three times per capita GDP, so it was considered as a dominated method compared with the control group.

**Table 3:** Comparing the cost-effectiveness of three methods of nutrition education in patients with T2DM.

	**Strategy**	**Cost (PPP$)**	**Effectiveness^**a**^**	**Incremental cost**	**Incremental effectiveness**	**ICER^**b**^ (Incremental cost per extra success) PPP$**
Cost- effectiveness analysis (CEA)	Control group	364	0.40	0	0	0
	Web-tel education	5,335	0.63	4,971	0.23	21613.04*
	Group education	27,188	0.46	26,824	0.06	447,067 **

*^a^Effectiveness refers to reduction in HbA1c level (%)*.

*^b^ICER, incremental cost effectiveness ratio*.

^*^One to three times threshold for Iran in 2021 was ($13,116 PPP-$39,348 PPP): The ICER was less than three times per capita GDP.

^**^One to three times threshold for Iran in 2021 was ($13,116 PPP-$39,348 PPP): The ICER was more than one to three times per capita GDP.

**Figure 3:**
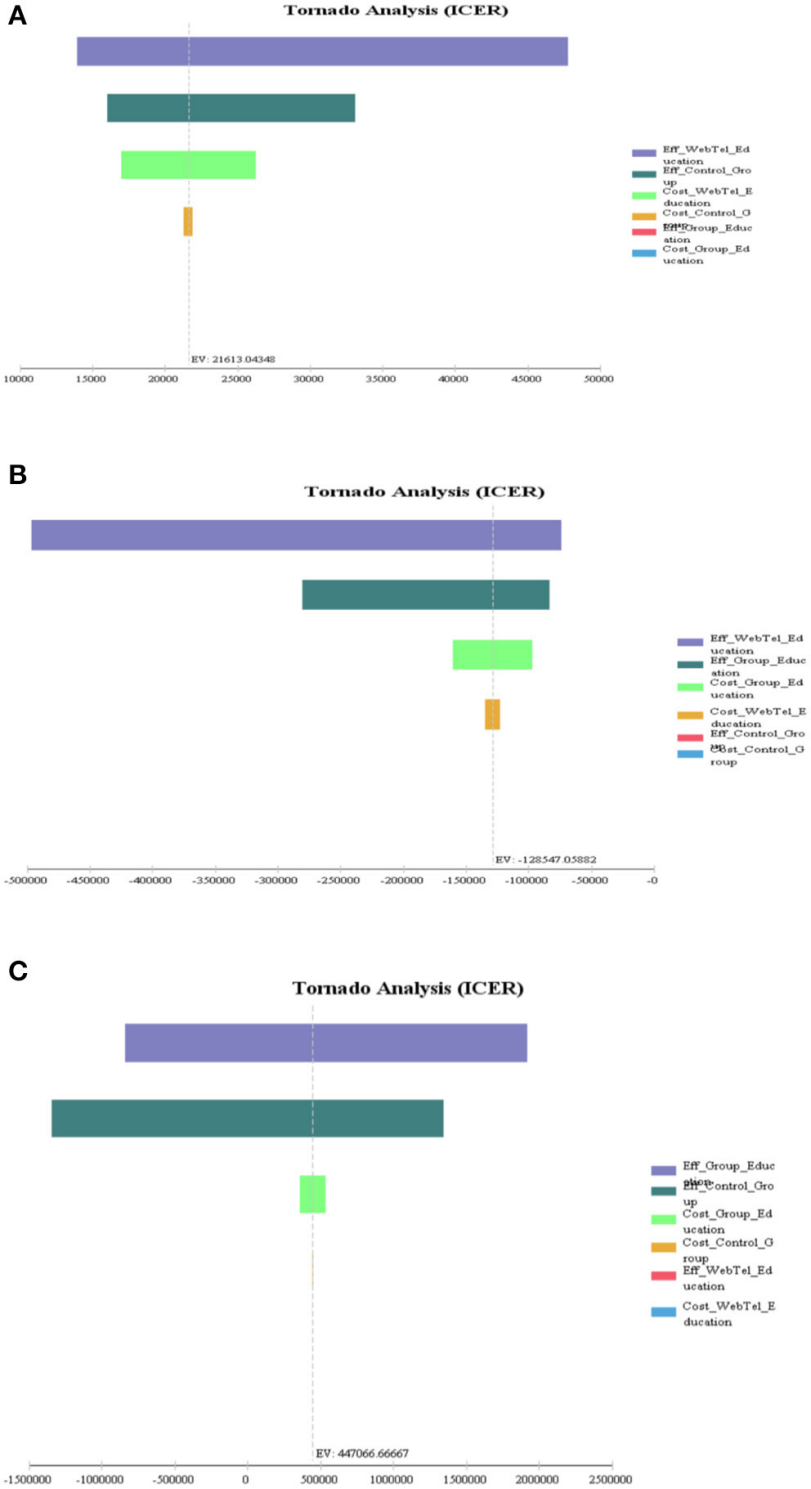
One-way sensitivity analysis and tornado diagram of incremental cost-effectiveness ratio (ICER) comparisons among the three groups of patients with type 2 diabetes. **(A)** Web-Tel education vs. control group, **(B)** Web-Tel education vs. group education, and **(C)** group education vs. control group.

The results of the tornado diagram showed that in the comparison of group education vs. control group, ICER results had the most sensitivity to the effectiveness of group Education and the control group; and in the comparison of the Web-Tel education vs. control group, the model was most sensitive to the effectiveness of Web-Tel education. Also, in comparing Web-Tel education with group education, ICER results had the highest sensitivity to the effectiveness of Web-Tel education (Figure 3) (16).

## Discussion

According to our results, the web-based education was more effective in reducing HbA1c level than group education or the control group. It was compatible with the results of another study conducted on 40 patients with T2DM for 6 months which showed that applying a web-based education system could be more effective than traditional education for glycemic control in patients with diabetes (17). Also, in agreement with the cost-effectiveness results of the present study, Li et al. (13) (in the United Kingdom), compared a 1-year web-based self-management training method with usual care for 374 patients with T2DM. The results showed that the web-based training was considered as a cost-effective method, equal to £20,000 to 30,000 per unit of quality of life (13). Another study in 2014 assessed the cost-effectiveness of an American community-based educational program for patients with type 2 diabetes. They found that the intervention improved HbA1c and QALY significantly by 0.056 per person (per unit of quality of life equivalent to $ 355) compared to the control group (12). Conversely, a 1-year randomized clinical trial (in 2015) on the cost-effectiveness of group education in patients with diabetes showed that the clinical outcome was reduced significantly in the intervention group compared to the control group who received usual care. The quality of life was also improved by 0.067, equal to 1,862 US dollars per person (11).

The observed controversy between the findings of the present study and previous ones can be justified by the following facts. In the present study, the problems reported by patients who attended in group education classes were lack of time and distance problem. Moreover, because of the COVID-19 pandemic and its high pathogenicity among the elderly and those with weak immune system, the group education method was not welcomed by the patients with diabetes. Although the groups were small, the patients were still concerned about the high transmissibility of the virus, especially in a situation where minority of the population got the first dose of vaccine, and the vast majority of the population was unvaccinated. Another reason that group education dominated the other groups/methods is probably the fact that the educational content was not accessible at any time. Therefore, virtual education such as Web-Tel education, with lower cost and higher effectiveness, can be a suitable alternative for group education to meet the needs of patients with T2DM. The Web-Tel method enables the participants to access information at any time and without the stress of participating in group classes. Moreover, based on the participants' opinion, this method was simple, comfortable, and time-saving.

Considering the fact that a considerable part of the costs of patients with diabetes is related to direct costs, which account for 2.5 to 15% of the total health budget in different countries (18), the present study aimed to investigate the direct medical costs of patients with diabetes. In Iran, a significant amount of costs of patients with diabetes are covered by health insurance. Also, education of patients with diabetes is performed in health centers and is covered by healthcare providers for free. However, three issues must be considered to justify the patients' perspective in the present study. First, previous studies have shown that despite health insurance coverage, the cost that have to be paid directly by patients is still high in Iran and other developing countries, which have faced the healthcare system with serious challenges (19, 20). Second, nutrition education in health centers does not cover many special nutrition concepts such as counting carbohydrates or traffic light labels of foods (21). In addition, the education of patients with diabetes in health centres has been limited due to the COVID-19 pandemic. In fact, many centres refused to provide these services for patients with diabetes as a high-risk population for Corona virus. The last two issues forced people with diabetes to refer to nutrition clinics to get nutrition education where the entire cost was covered by the patients. So, in the present study the patients' perspective was selected to investigate the cost-effectiveness of nutrition education in patients with diabetes, that the costs are totally or to a large extent covered by the patients.

The present study has some strengths. This is the first study that assessed the effectiveness and cost-effectiveness of two different nutrition educational methods in patients with T2DM, especially in Iran. The other two strengths include, creating active environment and using text-messages/calls during trial, to become aware of receiving educational contents by the participants and minimizing their lost to follow up. On the other hand, it has some limitations. First, because of the COVID-19 pandemic, group education was held with few participants in each class (maximum of six), so the limited number of participants decreased the interactions and class discussions. Second was the short duration of the study of about 3 months. Third was detection of a large amount of uncertainty associated with the results according to the sensitivity-analysis. Fourth and last is the generalizability of the findings. Nutrition education in Iran is used for the treatment of patients with T2DM, and its price is the same throughout the country, so the results of this study can be generalized to the whole country. However, because of differences in thresholds and prices, the results may not be comparable to other parts of the world; thus, the results should be interpreted with caution.

The results of the present study suggested the use of Web-Tel education as a first priority in educating patients with T2DM to reduce the financial burden in the community. The Web-Tel education method reduced the costs and increased the clinical outcomes in comparison with the group education method or the control group. On the other hand, group education had a high cost with low effectiveness, which indicated it to be a dominated method compared with the control group or the Web-Tel method for educating patients with T2DM. It must be mentioned that the results were highly sensitive to several key inputs; therefore, they must be interpreted with caution.

## Data Availability Statement

The raw data supporting the conclusions of this article will be made available by the authors, without undue reservation.

## Ethics Statement

The studies involving human participants were reviewed and approved by research Ethics Committee of Shiraz University of Medical Sciences, Shiraz, Iran [IR.SUMS.REC.1399.1162]. The patients/participants provided their written informed consent to participate in this study.

## Author Contributions

SF helped in design conduction and coordination of the study materials. S-MD-R reviewed the literatures, designed the protocol, conducting the intervention, and wrote the manuscript. KK and DG cooperated in the protocol design, data analysis, and calculating cost-effectiveness. SF, KK, and DG revised the manuscript. SF had primary responsibility for the final content. All authors read and approved the final manuscript.

## Funding

The present study sponsored by Vice Chancellor for Research, Shiraz University of Medical Sciences. Contact information: 071-32357282.

## Conflict of Interest

The authors declare that the research was conducted in the absence of any commercial or financial relationships that could be construed as a potential conflict of interest.

## Publisher's Note

All claims expressed in this article are solely those of the authors and do not necessarily represent those of their affiliated organizations, or those of the publisher, the editors and the reviewers. Any product that may be evaluated in this article, or claim that may be made by its manufacturer, is not guaranteed or endorsed by the publisher.

## Footnotes

^1^ http://databank.worldbank.org/data/reports.aspx?source=2&series=NY.GDP.PCAP.PP.CD&country=BWWDIAf

^2^ https://databank.worldbank.org/home.aspxBWWDIAf
