# Supplementary material for: Cost-Effectiveness Analysis of Group vs. Weblog Telecommunication (Web Tel) Nutrition Education Program on Glycemic Indices in Patients With Non-Insulin Dependent Diabetes Mellitus Type 2: A Randomized Controlled Trial
Source: Front Nutr. 2022 Jun 24;9:915847. doi: 10.3389/fnut.2022.915847 (PMC9270004; doi:10.3389/fnut.2022.915847)
Supplement: Supplementary file 1 [file Table_1.docx]

**Table S1.** Educational content of both: group and Web-Tel education.

| **Class** | **Educational Contents** |
| --- | --- |
| First Session | - Teaching healthy eating concept according to diabetic healthy eating guidelines - Teaching portion sizes by using pictures and models |
| Second Session | - Review on the previous session content - Teaching Glycemic index (GI) and glycemic load (GL) of foods - Teaching the effect of energy and macronutrients intake on body weight and blood glucose control - Teaching different types of dietary fats and their impacts on health |
| Third Session | - Review on the previous session content - Teaching carbohydrate counting - Teaching what is food label and how to apply it - Teaching what is traffic light on foods and how to apply it |
