# Supplementary figures and images for: Cost-Effectiveness Analysis of Group vs. Weblog Telecommunication (Web Tel) Nutrition Education Program on Glycemic Indices in Patients With Non-Insulin Dependent Diabetes Mellitus Type 2: A Randomized Controlled Trial
Source: Front Nutr. 2022 Jun 24;9:915847. doi: 10.3389/fnut.2022.915847 (PMC9270004; doi:10.3389/fnut.2022.915847)

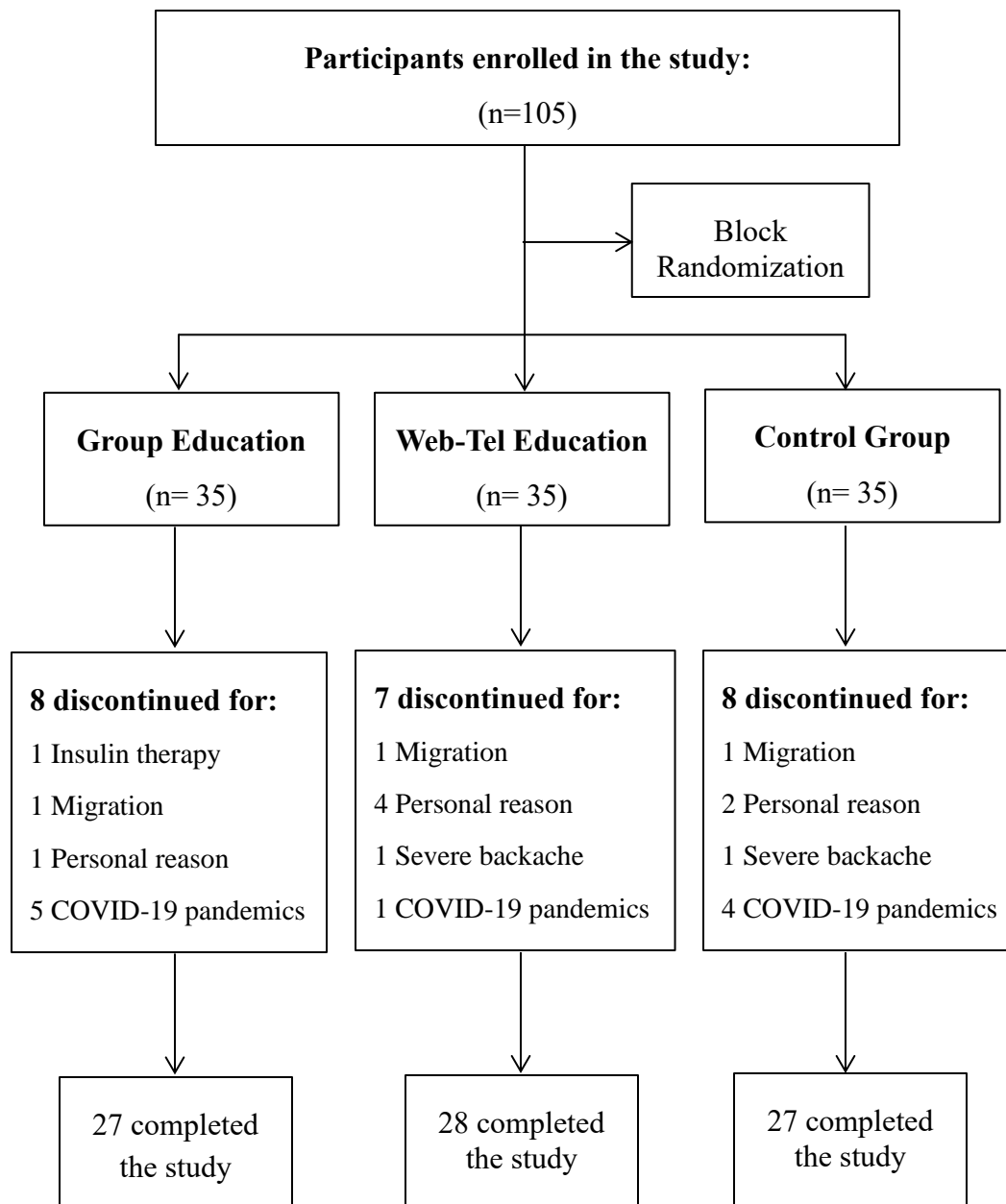

**Fig. S1.** Flowchart of study participants

Supplement: Supplementary file 2 [file Image_1.pdf]
